# Supplementary material for: Content-rich biological network constructed by mining PubMed abstracts
Source: BMC Bioinformatics. 2004 Oct 8;5:147. doi: 10.1186/1471-2105-5-147 (PMC528731; doi:10.1186/1471-2105-5-147)
Supplement: Additional File 5 — The original Chilibot query results of the term "long-term potentiation (LTP)" and 22 other terms, limiting the latest references analyzed to the years 1990, 1995, 2000, and 2004. [file 1471-2105-5-147-S5.bz2 › chilibotAdditionalFile5/ltp2000/html/PKA_ACTIN.html]

 


 **PKA** and **ACTIN** 
  
Found 56 abstracts in PubMed,  **30 abstracts were retrieved and analyzed**.  


---

 Search Google  |
 PDF files only 
|  EDU domain only 

---

**Interactive relationship** (e.g. stimulation, inhibition, etc)

- **PKA**  activating agents inhibited  **actin**  stress fiber formation and stretching of cells at the wound margin.  Ref: 9834339 J Gastrointest SurgJ Gastrointest Surg, 1999
- Inhibition of protein kinase A  **PKA** ... totally blocked the induction of  **actin**  mRNA by T3.  Ref: 10101242 Brain Res Mol Brain Res, 1999
- The following conclusions were drawn 1 In excisional wounds of hepatocyte monolayers, both EGF and  **PKA**  exert action on  **actin**  microfilaments, which are stretched by EGF and inhibited by  **PKA** .  Ref: 9834339 J Gastrointest SurgJ Gastrointest Surg, 1999
- We demonstrate that RhoAV14A188, which cannot be phosphorylated by  **PKA**  in vitro, is more effective than RhoAV14 in preventing cells from responding to cAMP and in inducing  **actin**  stress fiber formation.  Ref: 9712882 J Biol Chem, 1998
- The involvement of  **PKA**  in mediating the induction of  **actin**  mRNA by T3 was confirmed by transfecting primary cultures of rat cerebra with an expression vector encoding the protein kinase A inhibitor which totally abolished the induction.  Ref: 10101242 Brain Res Mol Brain Res, 1999
- NT 3 treatment resulted in a rapid and transient stimulation of  **PKA**  activity that preceded the localization of beta  **actin**  mRNA.  Ref: 10508855 J Cell Biol, 1999
- 2 the  **PKA**  inhibitor H89 reversed the inhibitory effects of NECA on the  **actin**  dynamics.  Ref: 9548370 Eur J Cell Biol, 1998
- We conclude that a the functional properties of the cAMP activated NSC channel are similar to those of the stretch activated one, b the  **actin**  cytoskeleton plays a crucial role in the activation of the NSC channel induced by stretch and cAMP, and c the basal activity of the NSC channel is maintained by  **PKA**  dependent phosphorylation but is not dependent on  **actin**  microfilaments.  Ref: 9276757 J Gen Physiol, 1997
- The following observations indicate that the A2 receptor induced effects involve the cAMP protein kinase A  **PKA**  signaling pathway 1 a combination of NECA and the cAMP specific phosphodiesterase PDE inhibitor Ro 20 1724 raised the cAMP content in both unstimulated and stimulated neutrophils and also further inhibited the  **actin**  dynamics.  Ref: 9548370 Eur J Cell Biol, 1998
- ERM family members serve to anchor EBP50 and E3KARP to the  **actin**  cytoskeleton and sequester protein kinase A  **PKA**  to these protein complexes.  Ref: 11124833 Hepatology, 2001
- Thus, we propose a previously unrecognized function for WAVE 1 as an  **actin**  associated scaffolding protein that recruits  **PKA**  and Abl.  Ref: 10970852 EMBO J, 2000
- Immunocytochemical analyses in Swiss 3T3 fibroblasts suggest that the WAVE 1 kinase scaffold is assembled dynamically as WAVE,  **PKA**  and Abl translocate to sites of  **actin**  reorganization in response to platelet derived growth factor treatment.  Ref: 10970852 EMBO J, 2000
- Competition experiments suggest that the  **PKA**  WAVE 1 interaction may be regulated by  **actin**  as the kinase binds to a site overlapping a verprolin homology region, which has been shown to interact with  **actin** .  Ref: 10970852 EMBO J, 2000

**Parallel relationship** (e.g. studied together, co-existance, homology, etc.)

- All the results obtained strongly suggest that, in rat right ventricular trabeculae, beta adrenergic stimulation may increase the rate of cross bridge cycling by increasing the rate of cross bridge detachment from  **actin**  through a  **PKA**  mediated mechanism, although  **PKA**  reduces the calcium sensitivity of the contractile system.  Ref: 9220351 J Mol Cell Cardiol, 1997
- These results suggest that, in rat right ventricular trabeculae, beta adrenergic stimulation may increase the rate of cross bridge cycling by increasing the rate of crossbridge detachment from  **actin**  through a  **PKA**  mediated mechanism, although  **PKA**  reduces the calcium sensitivity of the contractile system.  Ref: 9889858 Adv Exp Med Biol, 1998
- Activators of  **PKA** ... potentiated the T3 induced  **actin**  gene expression, both at mRNA and protein level, by about 2 fold.  Ref: 10101242 Brain Res Mol Brain Res, 1999
